# Baculovirus-mediated gene transfer in butterfly wings *in vivo*: an efficient expression system with an anti-gp64 antibody

**DOI:** 10.1186/1472-6750-13-27

**Published:** 2013-03-25

**Authors:** Bidur Dhungel, Yoshikazu Ohno, Rie Matayoshi, Joji M Otaki

**Affiliations:** 1 The BCPH Unit of Molecular Physiology, Department of Chemistry, Biology and Marine Science, University of the Ryukyus, Nishihara, Okinawa, 903-0213, Japan

**Keywords:** Butterfly wing, Gene transfer, Baculovirus vector, Green fluorescent protein, gp64, Virus infection, Antibody injection, Immunohistochemistry

## Abstract

**Background:**

Candidate genes for color pattern formation in butterfly wings have been known based on gene expression patterns since the 1990s, but their functions remain elusive due to a lack of a functional assay. Several methods of transferring and expressing a foreign gene in butterfly wings have been reported, but they have suffered from low success rates or low expression levels. Here, we developed a simple, practical method to efficiently deliver and express a foreign gene using baculovirus-mediated gene transfer in butterfly wings *in vivo*.

**Results:**

A recombinant baculovirus containing a gene for green fluorescent protein (GFP) was injected into pupae of the blue pansy butterfly *Junonia orithya* (Nymphalidae). GFP fluorescence was detected in the pupal wings and other body parts of the injected individuals three to five days post-injection at various degrees of fluorescence. We obtained a high GFP expression rate at relatively high virus titers, but it was associated with pupal death before color pattern formation in wings. To reduce the high mortality rate caused by the baculovirus treatment, we administered an anti-gp64 antibody, which was raised against baculovirus coat protein gp64, to infected pupae after the baculovirus injection. This treatment greatly reduced the mortality rate of the infected pupae. GFP fluorescence was observed in pupal and adult wings and other body parts of the antibody-treated individuals at various degrees of fluorescence. Importantly, we obtained completely developed wings with a normal color pattern, in which fluorescent signals originated directly from scales or the basal membrane after the removal of scales. GFP fluorescence in wing tissues spatially coincided with anti-GFP antibody staining, confirming that the fluorescent signals originated from the expressed GFP molecules.

**Conclusions:**

Our baculovirus-mediated gene transfer system with an anti-gp64 antibody is reasonably efficient, and it can be an invaluable tool to transfer, express, and functionally examine foreign genes in butterfly wings and also in other non-model insect systems.

## Background

Diverse color patterns of butterfly wings are excellent two-dimensional systems to investigate development and evolution of pattern formation [1]. Eyespots are the most conspicuous color patterns and are composed of simple circular arrangements of colored scales. Eyespots are found in many nymphalid butterflies, including the blue pansy *Junonia orithya*, the buckeye *Junonia coenia*, and the squinting bush brown *Bicyclus anynana*, which are frequently used for experimental manipulations in butterfly biology. The developmental mechanism of eyespots in nymphalid butterflies has been studied using various methods, including surgical manipulations [2-6], physiological treatments [7], *in situ* hybridization histochemistry and immunohistochemistry [8-14], and morphological color pattern analysis [15-17]. The expression patterns of candidate regulatory genes for color pattern formation, such as *Distal-less*, *notch*, *engrailed*, *hedgehog*, *cubitus interruptus*, *patched*, and *spalt*, in *B. anynana* and *J. coenia* resemble a part of adult butterfly eyespots, suggesting their roles in eyespot formation [8-12,18,19]. A recent addition to this list is *Antennapedia*, which showed the earliest and exclusive expression in prospective eyespot foci in *B. anynana*[20].

However, there is no direct evidence of the roles of any candidate genes in eyespot formation due to a lack of reproducible and reliable functional assay systems. Random mutagenesis experiments did not produce diverse phenotypes in *B. anynana*[21], and therefore, spontaneous mutants are still the best way to analyze eyespot development in this species [22,23]. Germline transformation in butterflies has been reported [24], but its practicality is not entirely apparent, despite its labor-intensiveness. Likewise, the use of *in vivo* DNA electroporation has been limited due to physical damage to the wings caused by the procedure [25]. In contrast, viral vectors have been employed to transfer and express foreign genes in lepidopteran insects. Recombinant Sindbis virus vectors have been used to study the homeotic gene *Ultrabithorax* (*Ubx*) during the development of *J. coenia*[26], but most likely because it is an RNA virus, the use of Sindbis virus vectors has not been pursued further. Similarly, using vaccinia virus vectors has been reported, but high levels of expression have not been achieved [27].

Baculovirus vectors seem to be promising, as they infect butterflies in natural environments. Recombinant baculovirus vectors are simple, safe, and inexpensive to engineer, can infect various cell types, and have a large capacity for DNA inserts [28-30]. Recombinant baculovirus vectors have been used for ectopic expression in *Drosophila melanogaster*, the flour beetle *Tribolium castaneum*[31] and the silkworm *Bombyx mori*[32,33].

To our knowledge, the use of baculovirus vectors for gene transfer *in vivo* has not been reported in butterflies, which may largely be due to the high cytotoxic effects of baculovirus vectors in butterflies. Baculovirus-infected cells are known to undergo apoptosis as a part of defense mechanisms [34-36]. However, it may be possible to reduce the cytotoxic effects and develop a relatively efficient gene delivery method to butterfly wings using recombinant baculovirus vectors. Baculovirus-associated cytotoxic effects may originate from concentrated infection and subsequent cell death. To minimize these unwanted effects, we administered an anti-gp64 antibody. This antibody was raised against baculovirus coat protein gp64, an envelope protein that play a role in the cell-to-cell transmission of infection [37]. We hypothesized that this antibody may prevent unnecessary and excessive infection by baculovirus in developing cells. Using this “immunotherapy” for infected individuals, we successfully achieved a high survival rate after infection and high-level expression of a foreign gene (in this case, a gene for green fluorescent protein, GFP) in butterfly wings *in vivo*.

Methodologically, pupae have been shown to be highly resistant to chemical injection [7,38-40], which has contributed to our understanding of color pattern determination in butterfly wings. In this paper, we employed microsyringe-assisted injection as a simple delivery method for baculovirus vector and antibody. We demonstrated that pupae were resistant to injections twice at the same injection point. This resistance against double chemical injections allowed us to establish a reliable gene delivery system in butterfly wings.

## Methods

### Butterflies

Throughout this study, we used the blue pansy butterfly *J. orithya* (Linnaeus, 1758). Female adult individuals were caught in Okinawa-jima Island or Ishigaki-jima Island in the Ryukyu Archipelago, Japan, and eggs were collected from these females. Alternatively, larvae were field caught in these islands. Larvae were fed their natural host plants at ambient temperature.

### Baculovirus vector, anti-gp64 antibody, and injection

Recombinant baculovirus vector containing the *Aequorea victoria* green fluorescent protein (GFP) gene under the control of the polyhedrin promoter was obtained from AB Vector (San Diego, CA, USA) at a viral titer of 1 × 10^8^ pfu/mL. In the present study, we expressed titers using two digits based on dilution factors, but only one digit is significant, as in the non-diluted original titer. A mouse monoclonal IgG_2a_ antibody against baculovirus gp64 (AcV1) of extracellular nonoccluded AcNPV (*Autographa californica* nucleopolyhedrovirus) (200 μg/mL in PBS) was obtained from Santa Cruz Biotechnology (Santa Cruz, CA, USA). In the case of the 2.0-μL injection, pfu/mL can be converted to pfu/individual by the factor of × (2 × 10^-3^). Pupae were injected with 2.0 μL (unless otherwise specified) of a solution containing the baculovirus in the cuticle of the abdomen within 24 hours after pupation, followed by antibody injection at the same position at various volumes and times, as indicated, using an Ito microsyringe (Fuji, Shizuoka, Japan).

### Visualization of the GFP fluorescent signal

Whole pupae, whole adults, isolated pupal wings, and isolated adult wings were placed on an ATTO illuminator VISIRAYS-B (Tokyo, Japan), a blue LED light unit with emission wavelengths λ = 440–500 nm and λ_max_ = 470 nm. GFP fluorescence was observed at low magnification with this illuminator, and images were recorded using the digital single-lens reflex camera Canon EOS 50D (Tokyo, Japan) with the ATTO filter SCF515. We used the following imaging system for high-magnification images of GFP fluorescence: a Nikon inverted epifluorescence microscope Eclipse Ti-U (Tokyo, Japan) equipped with a Nikon Intensilight C-HGFI (a mercury pre-centered fiber illumination system), a Coherent Sapphire 488 nm laser generator (Santa Clara, CA, USA), a Yokogawa Electric CSU-X1 confocal scanner unit (Tokyo, Japan), and a Hamamatsu Photonics ImagEM EM-CCD camera (Hamamatsu, Japan). This microscope hardware system was controlled with the Hamamatsu Photonics AQUACOSMOS 2.6 analysis system. We used a Nikon GFP-B fluorescent cube (excitation filter: 460–500 nm, dichroic mirror: 505 nm, and emission filter 510–520 nm) for GFP detection. When confocal images are presented, they are designated as such in this paper. For bright-field low-magnification images, we used the digital single-lens reflex camera Canon EOS 50D (Tokyo, Japan) and Saitou Kougaku SKM-S30-PC (Yokohama, Japan). For bright-field high-magnification images, we used the Keyence high-resolution digital microscope VHX-1000 (Osaka, Japan) and the Nikon microscope system described above.

### Degrees of GFP fluorescence in pupae

After the injection of the baculovirus vector, the treated pupae were evaluated for fluorescence every day using the ATTO illuminator VISIRAYS-B. We visually classified the level of GFP fluorescence in the GFP-positive pupae into three categories, GI, GII, and GIII. In G0, no fluorescence was observed in wings. In GI, GFP fluorescence was observed in less than 50% of the pupal forewing area (right or left wings). In GII, approximately 50% or more of the pupal forewing area (right or left wings) was covered with GFP fluorescence. In GIII, almost the entire pupal wing area (and often other parts of the body) was covered with GFP fluorescence. When GFP fluorescence was observed for the first time, that pupa was classified into one of the three categories (GI, GII, or GIII) based on visual inspection. The fluorescence level could be higher (if alive) or less (if dead) on subsequent days, but we did not evaluate these subsequent changes in fluorescent levels. Pupae were examined for five days after the injection of the baculovirus vector, and if we did not observe any GFP fluorescence, that pupa was defined as G0 (no fluorescence or GFP-negative).

### Pupal wing dissection and immunohistochemistry

Pupal wings from four-day-old pupae after pupation were dissected according to a published protocol, with some modifications [41]. The pupa was lightly anesthetized on ice, and the cuticle around the wing margin was cut using a scalpel and lifted to cut through the trachea connecting the wings to the thorax. Dissected wing tissues were placed on glass slides. The tissues were then directly subjected to the fluorescent microscope to examine the GFP fluorescence. They were then air dried and stored in a refrigerator until use for immunohistochemistry.

For the immunohistochemical detection of GFP in pupal wings, we followed a modified protocol of previous studies [11,42]. We used monoclonal anti-GFP antibody (mouse IgG_2b_, clone 1E4) raised against recombinant full-length GFP (246 amino acids) (Medical & Biological Laboratories, Nagoya, Japan) as the primary antibody at a 1:200 dilution in the following solution: 50 mM Tris (pH 6.8), 150 mM NaCl, 0.5% NP40, and 1 mg/mL BSA. For negative controls, we used a non-specific normal mouse IgG (Santa Cruz Biotechnology) at a 1:200 dilution in the same solution. After incubating the dissected wings with anti-GFP antibody or normal mouse IgG, the wings were treated with secondary anti-mouse IgG antibody (Santa Cruz Biotechnology) in phosphate-buffered saline (PBS) and the VectaStain Elite ABC Kit (Vector Laboratories, Burlingame, CA, USA). For chromogenic detection, the DAB (3,3^′^-diaminobenzidine) Substrate Kit for Peroxidase was employed (Vector Laboratories). The wings were mounted in Softmount (Wako, Osaka, Japan), and pictures were taken using a Keyence high-resolution digital microscope VHX-1000 (Osaka, Japan).

## Results

### GFP fluorescent signals in pupae

**Figure 1:**
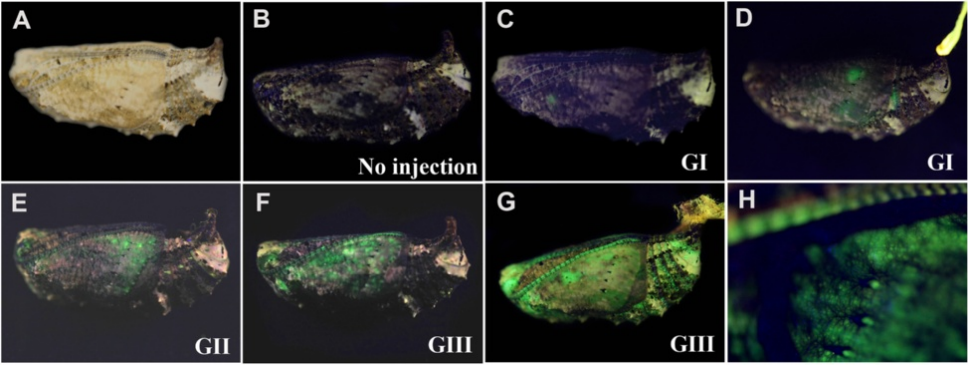
**Baculovirus-mediated GFP expression in *J. orithya *pupae.** Baculovirus was injected at the pupal stage 6–12 hours after pupation. (**A**) A whole pupa under the bright field showing the GI level. The injection consisted of 2.0 μL at 2.5 × 10^2^ pfu/mL. This individual is identical to C. (**B-G**) Whole pupae under blue light showing various GFP fluorescent levels. (**B**) G0. No injection (normal pupa) (**C**) GI. (**D**) GI. The injection consisted of 2.0 μL at 5.0 × 10^5^ pfu/mL. (**E**) GII. The injection consisted of 2.0 μL at 2.0 × 10^5^ pfu/mL. (**F**) GIII. The injection consisted of 2.0 μL at 5.0 × 10^5^ pfu/mL. The level of GFP fluorescence varied even if the same conditions were used in D and F. (**G**) GIII. The injection consisted of 2.0 μL at 1.0 × 10^7^ pfu/mL. (**H**) Higher magnification of the wing region of the fluorescing pupa shown in D.

We injected the GFP-containing baculovirus vector at various titers into the segmental boundaries of pupae 6-12 hours after pupation. Although the vector was injected into either the right or left side of the abdomen, GFP epifluorescence was observed in various parts of the pupal body. The degree of fluorescence varied, and infected sections were heterogeneous among individuals (Figure 1A-H). In some individuals, the whole body fluoresced, including wings, antennae, eyes, abdomen, and proboscis (Figure 1G). It appeared that wings were one of the tissues that showed GFP fluorescence relatively frequently. This result may be simply because wings occupy a relatively large surface area in a pupa. In other individuals, there was no sign of fluorescence at all (not shown). The autofluorescence in non-infected individuals was virtually negligible (Figure 1B), and when present, it was yellowish (not shown), which was easily distinguishable from the green fluorescence of GFP. We never observed GFP-like green fluorescence in non-infected individuals (*n* > 10).

**Figure 2:**
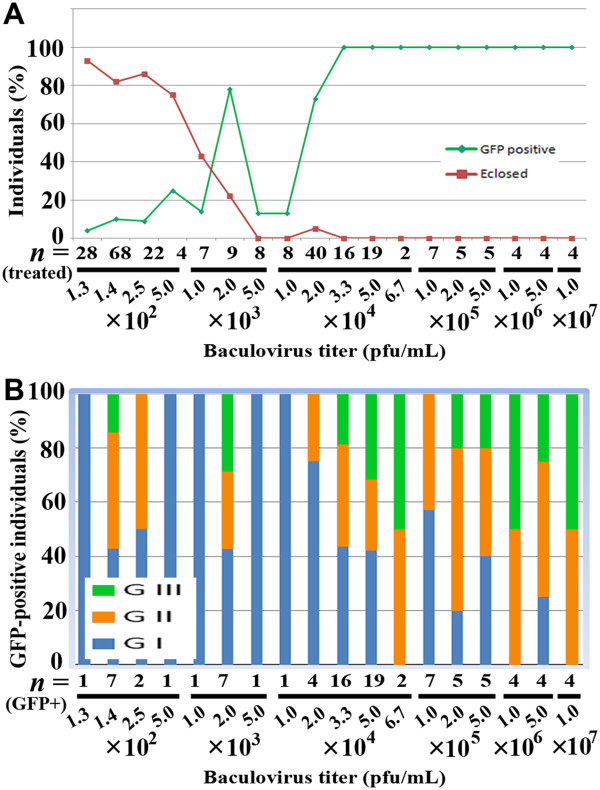
**Percentages of GFP-positive individuals, eclosed individuals, and the degrees of fluorescence in baculovirus-injected *J. orithya *pupae.** Virus titers are shown in pfu/mL. Note that pfu/mL can be converted to pfu/individual by the factor of × (2 × 10^-3^). (**A**) Percentages of GFP-positive pupae (green) and successfully eclosed individuals (red) at various baculovirus titers. Baculovirus (2 μL) was injected 24 hours post-pupation. (**B**) Proportions of GI, GII, and GIII levels of GFP fluorescence in fluorescent pupae at various baculovirus titers.

At high baculovirus titers (3.3 × 10^4^ pfu/mL and higher), the percentage of GFP-positive individuals was 100%, and at lower virus titers, it was as low as 4% (Figure 2A). A sudden transition between high and low percentages of GFP-positive individuals was detected between 1.0 × 10^4^ pfu/mL (13%) and 3.3 × 10^4^ pfu/mL (100%). Although an unexpected peak was observed at 2.0 × 10^3^ pfu/mL, this could be due to sensitivity variations of larvae or technical inconsistency in the injection procedure. In contrast to the high percentages of GFP-positive individuals at high titers, the percentages of eclosed individuals were very low at 5.0 × 10^3^ pfu/mL or higher. At lower titers, the percentages of eclosed individuals were higher, with low percentages of GFP-positive individuals.

We observed various levels of GFP fluorescence in the treated pupae, including G0 (Figure 1B), GI (Figure 1C, D), GII (Figure 1E), and GIII (Figure 1F,G). As expected, the GI category was mostly observed at lower titers, whereas the GII and GIII categories were more frequently observed at higher titers (Figure 2B).

Taken together, we demonstrated that the injection of baculovirus vector can deliver a foreign gene (i.e., GFP reporter gene) to butterfly pupae. However, no GFP-positive pupae eclosed or developed color patterns, which means that pupal development was halted by the lethal effects of the baculovirus vector. Pupae turned black and tissues were liquefied inside the pupal case, suggesting that baculovirus-infected cells underwent apoptosis, leading to an organismal death (not shown). We failed to identify titers that were suitable for both GFP expression and eclosion or at least color pattern development. In other words, simply reducing virus titers could not produce optimal conditions for gene transfer.

### Antibody treatment increased the survival rate of infected pupae

The major problem of using a baculovirus vector was the high pupal mortality rate associated with infection. To circumvent this problem, we used an anti-gp64 antibody. To evaluate the effect of this antibody, we used a titer of 2.0 × 10^4^ pfu/mL, which was in the transition region between high and low percentages of GFP-positive individuals, in subsequent experiments (see Figure 2A).

**Figure 3:**
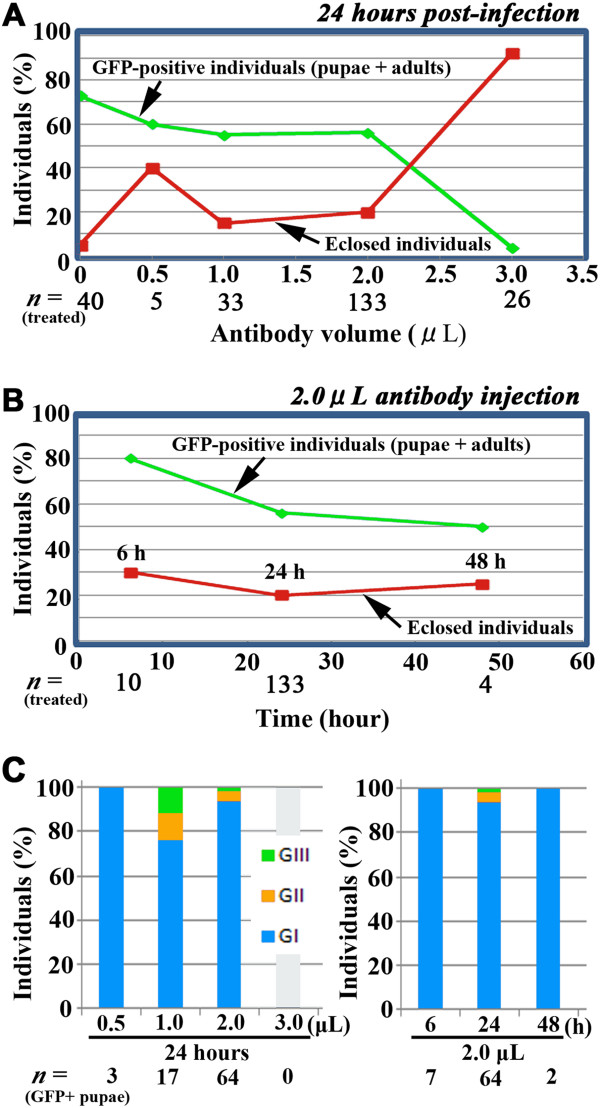
**Effects of the anti-gp64 antibody.** Baculovirus vector was used at 2 × 10^4^ pfu/mL . (**A**) Percentages of GFP-positive individuals and eclosed individuals at various antibody volumes. The antibody treatment was performed 18–24 hours post-infection. (**B**) Percentages of GFP-positive individuals and eclosed individuals at various time points of antibody treatment (6 hours, 24 hours, and 48 hours post-infection). The antibody volume used was 2.0 μL. (**C**) Degrees of GFP fluorescence (GI, GII, and GIII) in GFP-positive individuals. The left panel shows the results of various antibody volumes 18–24 hours post-infection, and the right panel shows the results of various treatment times using 2.0 μL antibody.

**Figure 4:**
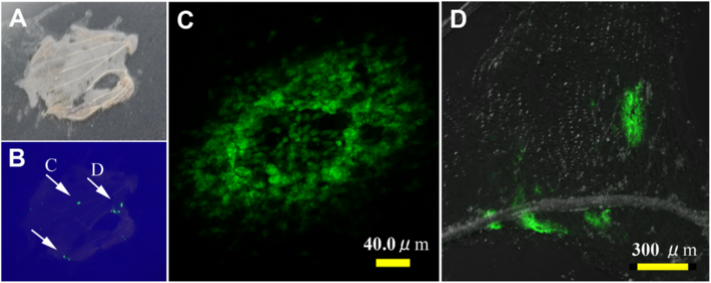
**GFP fluorescence in a developing *J. orithya *pupal wing at the cellular level.** The baculovirus vector was injected at 1.0 × 10^6^ pfu/mL (2.0 μL) 18–24 hours post-pupation, followed by anti-gp64 antibody injection (2.0 μL) 18–24 hours post-infection. Pupal wings were dissected 24 hours post-antibody treatment. (**A**) A whole wing under the bright field. (**B**) A whole wing identical to A under blue light. Three major fluorescent clusters are indicated by arrows, two of which are shown in C and D. (**C**) Confocal image of a fluorescent region shown in B. A circular arrangement of the fluorescent signals is seen. (**D**) Confocal image of another fluorescent region shown in B, superimposed on the bright field picture.

**Figure 5:**
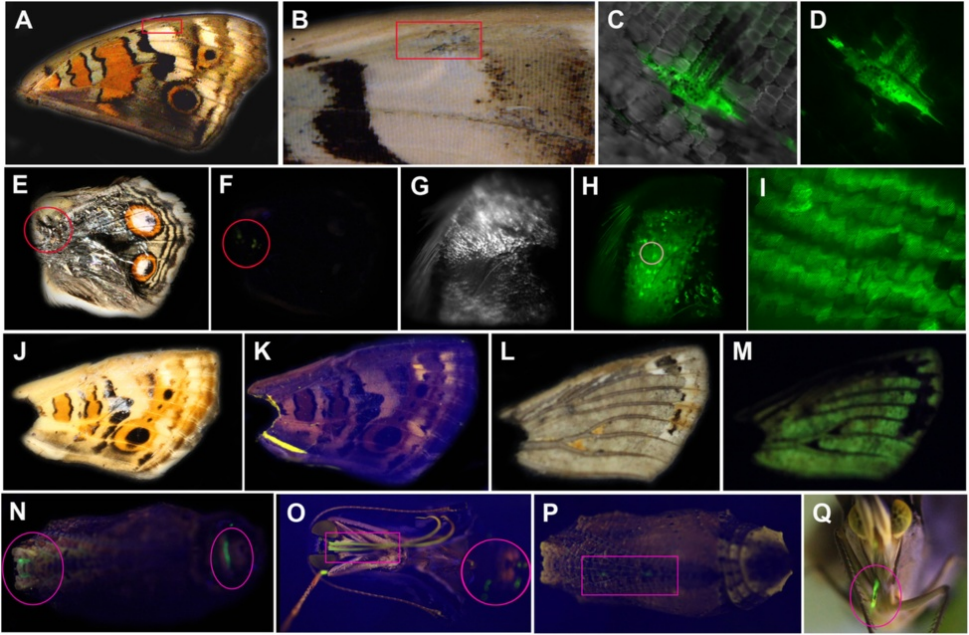
**Baculovirus-mediated GFP expression in *J. orithya *pupae and adults obtained from baculovirus-injected and antibody-injected individuals.** (**A**) An adult ventral forewing. The boxed region is enlarged in B. (**B**) High magnification of A. The boxed region is enlarged in C and D. (**C**) High magnification of B under blue light and a small degree of white light. Intense fluorescence was observed in regions where no scales were found, but some scales also fluoresced. (**D**) High magnification of C under blue light. (**E**) An adult dorsal hindwing, isolated from a pupal case due to eclosion failure. The circled region is enlarged in G and H. (**F**) The same wing in E under blue light. A few clusters of green fluorescence were observed, as indicated by circles. (**G**) High magnification of E. (**H**) The same region as G. The circled region is magnified in I. (**I**) High-magnification confocal image of H. Intensive fluorescence was observed in scales. (**J**) An adult ventral forewing, isolated from a pupal case due to eclosion failure. (**K**) The same wing as J under blue light. No green fluorescence was detected. (**L**) The same wing as J after the removal of scales. (**M**) The same wing as L, with scales removed, under blue light. Green fluorescence was observed throughout the wing. (**N**) A pupa with fluorescence in a section of the proboscis and abdomen (circled). The wing shown in E was obtained from this pupa. (**O**) An eclosed adult from the pupa in N. A section of the proboscis (boxed) and abdomen (circled) shows green fluorescence, as predicted at the pupal stage. (**P**) A pupa with green fluorescence in a part of a leg (boxed). (**Q**) An eclosed adult from the pupa in P. A part of a leg shows fluorescence, as predicted at the pupal stage.

We injected 0.5, 1.0, 2.0, and 3.0 μL of the antibody 18–24 hours after injection of the baculovirus vector (Figure 3A). With antibody injection and an increase in antibody volume, the percentage of GFP-positive individuals decreased from 73% to 4%, but the percentage of individuals that eclosed successfully increased from 5% to 92% (Figure 3A). Importantly, in the range of 0.5–2.0 μL, significant proportions of treated individuals successfully eclosed, with accompanying GFP fluorescence. These results demonstrated that “immunotherapy” with the anti-gp64 antibody rescued a significant proportion of infected pupae from death and, at the same time, reduced the percentage of GFP-positive individuals. Thus, it is possible to obtain GFP-positive wings with color pattern development completed (see Figures 4 and 5).

Fixing the injection volume at 2.0 μL, we injected the antibody at 6, 24, and 48 hours after baculovirus treatment. The percentage of GFP-positive individuals decreased from 80% to 50%, and the percentage of individuals that eclosed successfully remained in the range of 20–30% (Figure 3B). We classified the GFP-positive pupae into three categories, GI, GII, and GIII, based on the degrees of fluorescence (Figure 3C). Under the conditions of 2.0-μL or 1.0-μL antibody injection 24-hours post-infection, GII and GIII levels were obtained despite the relatively small proportions. Together, we conclude that 0.5–2.0 μL of the gp64 antibody solution delivered 6–24 hours after baculovirus treatment is the optimal condition for gene transfer and expression in pupae.

### GFP fluorescent signals in developing cells in wings

We used a confocal microscope to identify the source of fluorescence in developing wings from pupae treated with the baculovirus and the anti-gp64 antibody (Figure 4A-D). We examined 4 treated pupae and obtained 9 GFP-positive wings. After the isolation of wings from pupae two or three days post-pupation, a few fluorescent sections were found per wing in all cases, and fluorescent cells constituting a wing were observed. Most likely, they would differentiate into both socket and scale cells. We observed that the fluorescent cells occasionally formed a circular pattern, and its central region was relatively dark in all cases, which may be caused by the cytotoxic effects of the baculovirus infection.

### GFP fluorescent signals in adult tissues

Because of the therapeutic effects of the anti-gp64 antibody, we examined several individuals that developed color patterns inside the pupal case or successfully eclosed to become adults with GFP fluorescence in wings and other body parts. Among 13 individuals examined, 7 individuals showed GFP fluorescence in wings, and 6 individuals only showed fluorescence in other parts of the body. In one successfully eclosed wing obtained from a baculovirus-injected and antibody-injected (2.0 μL, 6 hours post-infection) individual (Figure 5A-D) and one fully developed wing in the pupal case obtained from a baculovirus-injected and antibody-injected (1.0 μL, 18–24 hours post-infection) individual (Figure 5E-I), we confirmed that fluorescence signals originated from the scales themselves (Figure 5D,I). In most cases, however, GFP fluorescence originated from the basal membrane (only when scales were removed) but not from scales (*n* = 5; Figure 5J-M; This wing shown here was obtained from a baculovirus-injected and antibody-injected [2.0 μL, 6 hours post-infection] individual). In two cases, GFP fluorescence was observed in the entire area of the wing basal membrane. We confirmed that green fluorescence was not detected at all in non-infected wings before and after scale removal (not shown). GFP fluorescent signals were also observed in eyes, antennae, palpi, the proboscis, and the abdomen (*n* = 6; Figure 5N-Q; The P-Q individual shown here is a baculovirus-injected and antibody-injected [2.0 μL, 18–24 hours post-infection] individual). Importantly, no aberrant change in color patterns was observed in the wings with GFP fluorescence (*n* = 7; Figure 5A,E,J).

### Immunohistochemical detection of GFP in pupal wings

**Figure 6:**
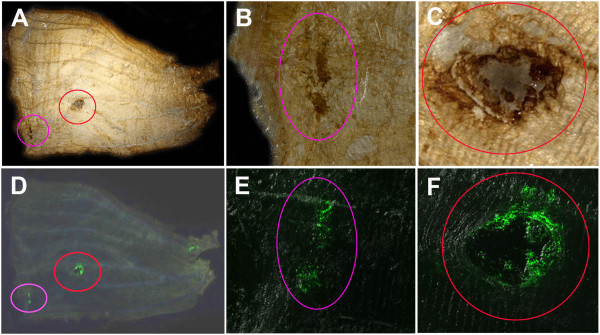
**Immunohistochemical detection of GFP in developing *J. orithya *pupal wings with anti-GFP antibody.** A fourth-day pupal wing infected with 2.0 × 10^4^ pfu/mL baculovirus vector (2.0 μL, 18-24 hours post-pupation) and treated with anti-gp64 antibody (2.0 μL, 18-24 hours post-infection) is shown. Immunohistochemical DAB staining and GFP fluorescent signals overlapped with each other. (**A-C**) Immunohistochemical DAB staining using anti-GFP antibody. Two major regions indicated by circles were stained in A, and they are magnified in B and C. (**D-F**) GFP fluorescence signals from the same wing in A-C. Two major regions indicated by circles showed fluorescence, and they are magnified in E and F.

To confirm that the green fluorescence we observed was not autofluorescence but indeed originated from ectopically expressed GFP, we performed immunohistochemical staining of pupal wing tissues using anti-GFP antibody. We allowed infected pupae to develop until the fourth day post-pupation, and the wings were then dissected and subjected to immunohistochemical staining. The chromogenic DAB signal for anti-GFP antibody (Figure 6A-C) and the GFP fluorescence (Figure 6D-F) from the same wing tissue completely overlapped in 4 wing samples from 4 different individuals. In these wing tissues, we observed that the GFP signals sometimes formed a circular structure, as seen in Figure 4. Its central region might have been damaged by baculovirus toxicity, although this circular pattern was not observed in the completed wings that were examined in Figure 5.

When we employed normal IgG instead of anti-GFP antibody with other procedures being the same, we detected no chromogenic DAB staining for GFP in the green fluorescent area in the 4 GFP fluorescence-positive wings from 2 individuals (not shown). These results demonstrated that the immunohistochemical DAB staining signals were not artifacts and that the green fluorescence in wings originated from the expressed GFP molecules.

## Discussion

In this study, we demonstrated that a recombinant baculovirus vector was able to deliver a foreign gene to wings of *J. orithya* pupae. Pupae treated with high titers of the baculovirus vector without subsequent antibody treatment showed GFP fluorescence but died at the pupal stage before color pattern development. This fact has most likely prevented researchers from pursuing the baculovirus-mediated gene transfer method in butterfly wings.

We first examined the GFP fluorescence levels at various virus titers. Heterogeneous GFP expression was observed at various levels in various parts of pupae even at the same titer, which could be due to individual variation (genetic heterogeneity) or random variation of the injection procedure (technical inconsistency). A similar type of infection variation has also been reported in other virus systems [43,44]. Nonetheless, we obtained dose-dependent changes in fluorescence. That is, at high baculovirus titers, we were able to obtain high percentages of GFP-positive individuals and the GIII level of GFP fluorescence. However, the high-level expression was accompanied by a high mortality rate. This is because of the induction of apoptosis in the infected cells [34-36]. Reducing virus titers did not allow the wing color patterns to develop with GFP fluorescence. This suggests that the minimum titer of baculovirus for GFP fluorescence can still induce cytotoxic effects sufficient to cause a pupal death. Therefore, the inhibition of baculovirus activity by anti-gp64 antibody is essential to obtain the color pattern development with GFP fluorescence.

To overcome the pupal death associated with the baculovirus infection, we used an antibody against the baculovirus coat protein gp64. Pupae tolerated double injection at the same injection point, and the therapeutic effect was dramatic. At high doses of anti-gp64 antibody, almost all pupae eclosed successfully. This result indicates that the anti-gp64 antibody prevented cells from dying due to infection with the baculovirus vector. Although high doses of the anti-gp64 antibody weakened the GFP expression level, we achieved eclosion rates of 10–40% and GFP-positive rates of 50–60% under optimized conditions. Examining GFP-positive individuals that completed color pattern development was possible with the administration of the anti-gp64 antibody. We do not know the precise mechanisms of how the immunotherapy with anti-gp64 antibody works. But it is likely that the antibody prevents further propagation of baculovirus by blocking the function of the coat protein gp64.

Mechanistically, the F_c_ receptor-mediated activation of immune cells, which is expected to work in mammals, may not be possible in insects because insects are not equipped with an adaptive immune system. Nonetheless, the binding of the anti-gp64 antibody to baculovirus particles appears to be sufficient to efficiently block the virus infection. This treatment is often sufficient to allow the infected individuals to completely metamorphose to adults. Using a mammalian antibody in insects for its therapeutic effect is most likely a new attempt. Additionally, the double injection of chemicals into a pupa is a new attempt. These new methodological aspects of this study may find applications in other fields of entomological research. For example, since a recombinant baculovirus vector is relatively easy to engineer, any non-model insects are now targets for the *in vivo* gene transfer experiments. Furthermore, this immunotherapy may be able to rescue insects of commercial use such as the silkworm naturally infected with baculovirus.

GFP-positive fluorescent cells were clearly detected in developing pupal wings. GFP expression was observed in not only wings but also other body parts, such as eyes and antennae. Broad tissue tropism was also reported in *J. coenia* using Sindbis virus [27] and in *Xenopus laevis* using baculovirus [31]. Interestingly, GFP fluorescence was observed in not only pupal wings but also adult wings. Although scales are dry extracellular structures, it appears that GFP molecules are able to be incorporated into scale structures. Importantly, GFP expression did not change the normal color patterns of wings.

The ability of baculovirus vectors to transfer foreign genes into developing wing tissues without any color pattern changes in adults makes this system useful in investigating gene functions in butterfly wing color pattern development *in vivo*. There are precedent cases of virus-mediated *in vivo* expression systems as functional assays of candidate genes in insects [26,31]. A non-insect example is the functional proof for an odorant receptor gene using an adenovirus-mediated gene transfer system [43,44]. We envision a similar *in vivo* functional assay system for the putative genes for color pattern formation in butterfly wings.

Recently, a relatively simple method to generate somatic transgenic cells in various insect tissues has been reported [45]. This electroporation method may be less toxic than our method, once a gene is integrated into host chromosome. Remarkably, the electroporation method achieved stable GFP expression in somatic cells in larvae and pupae [45]. However, the level of GFP expression in a pupal wing of the silkworm (Figure 2C in [45]) was much less than that of our study, and no expression in adult tissues was demonstrated. Furthermore, the electroporation method appears to be more technically demanding than our method. Each method has strength and weakness, and it is favorable that researchers will have an opportunity to try different methods suitable for an insect system of interest.

## Conclusions

The functional analysis of candidate genes for butterfly wing color pattern formation has been hampered by the lack of a method to manipulate gene expression. We have developed a method to transfer a foreign gene to pupal wings of *J. orithya* using recombinant baculovirus. We were able to express GFP in the developing wings of pupae by the simple injection of a recombinant baculovirus vector followed by a second injection with anti-gp64 antibody. The method developed here can be used for the functional study of candidate genes for wing color pattern development. The baculovirus vector in conjunction with the anti-gp64 antibody could also be an invaluable tool to investigate gene functions in other non-model insects.

## Competing interests

The authors declare that they have no competing interests.

## Authors’ contributions

JMO contributed to the conception and design of the experiments, analyzed the data, and wrote the manuscript. BD conducted the experiments, analyzed the data, and wrote the manuscript together with JMO. YO conducted the fluorescence microscopy for GFP. RM performed the immunohistochemical staining. All authors read and approved the final manuscript.
